# Effect of Steroids on the Biophysical Profile and Doppler Indices of Umbilical and Middle Cerebral Arteries in Preterm Fetuses

**DOI:** 10.7759/cureus.42334

**Published:** 2023-07-23

**Authors:** Swayamprava Behera, Sasmita Behuria, Jayashree J Moharana, Biranchi N Mohpatra, Rabindra Naik

**Affiliations:** 1 Department of Obstetrics and Gynecology, Srirama Chandra Bhanj Medical College & Hospital, Cuttack, IND

**Keywords:** preterm labor, middle cerebral arteries, umbilical arteries, doppler indices, biophysical profile, steroids

## Abstract

Introduction

Preterm birth is a major cause of perinatal death and disability and emerges as an important global public health problem. The antenatal administration of corticosteroids for preventing neonatal death due to respiratory distress syndrome and the serious morbidities associated with preterm birth has become an accepted standard in worldwide obstetric practice since 1994. After administering corticosteroids, the biophysical score deteriorates transiently, leading to a high cesarean section rate. Thus, Doppler indices of the umbilical and middle cerebral arteries are taken into account before the termination of pregnancy in preterm labor.

Materials and methods

This is an interventional study conducted over a period of one year and six months among 59 eligible singleton pregnancies at risk for preterm delivery, i.e., at risk of preterm birth <34 weeks of gestation, having no contraindication to antenatal steroids, who were admitted to the labor room of the Srirama Chandra Bhanja (SCB) Medical College, Cuttack, India, from January 2014 to August 2015. The participants were prospectively recruited, after giving informed consent to participate in the study. The main indication for hospital admission and steroid use was preterm lower abdominal pain.

Results

Out of 59 eligible cases, 27 (45.8%) were <25 years of age, 26 (44.1%) were between 25 and 30 years of age, and six (10.2%) were >30 years of age. The range is 14 years (between 19 and 33 years). The mean age is 25.31 years, with a standard deviation of 3.74. The mean of the biophysical profile (BPP) score before steroid administration decreased from 9.19 ± 1.23 to 5.56 ± 1.05 after 48 hours of first dose of steroid. The p-value of this is <0.001, which is statistically significant. The results show that the administration of betamethasone decreased fetal movements by 78.6% and breathing by 71.8%. As a result, the BPP scores decreased significantly. However, the Doppler indices did not change significantly even after administering corticosteroids.

Conclusion

Doppler indices play an important role in differentiating between steroid-induced compromise in the fetus and real fetal distress. Thus, umbilical and middle cerebral artery (MCA) Doppler should always be done before the termination of pregnancy on the ground of fetal compromise after administering corticosteroids.

## Introduction

The definition of preterm birth was first promulgated in 1976 by the World Health Organization (WHO) and the International Federation of Gynecology and Obstetrics (FIGO) [1]. Infants born between 34^0/7^ weeks and 36^6/7^ weeks experience morbidities and characteristic mortality; therefore, since 2005, preterm births have been subdivided into two categories [2]. Births before 33^6/7^ weeks are labelled early preterm, and those occurring between 34 and 36 completed weeks are called late preterm [3]. The antenatal administration of corticosteroids for the prevention of neonatal deaths due to respiratory distress syndrome and the serious morbidities associated with preterm birth has become an accepted standard in worldwide obstetric practice since 1994 [4].

Preterm birth is primarily a major cause of prenatal mortality, and impairment is considered a global health problem. Preterm birth is seen mostly in economically weak communities and those with high percentage of urinary and genital tract infections [5]. Insufficiency in neonatal healthcare facilities and expensive interventions, such as surfactant therapy, accelerate the problems of preterm births in low-resource settings [6]. Information on the efficacy of antenatal corticosteroids is relevant for these settings. This research is designed on a commentary in Cochrane reviews that sought to "assess the effects on fetal and neonatal morbidity and mortality, on maternal mortality and morbidity, and the child in later life of administering corticosteroids to the mother before anticipated preterm birth" and "assess the effects of different corticosteroid regimens for women at risk of preterm birth" [7].

In India, preterm births have reached approximately 3.6 million, accounting for 23.6% of global preterm births. In May 2012, the WHO and its partners - The Partnership for Maternal, Newborn and Child Health, Save the Children, and the March of Dimes - published a report titled “Born too soon: the global action report on preterm birth,” which included the first and initial estimate of preterm births in India [8]. "Born too soon" is the latest contribution to the UN Secretary-General's Global Strategy or Women's and Children's Health, which aims to save 16 million lives by 2030 [9].

## Materials and methods

An Interventional study conducted for one year and six months was performed on 59 eligible single-fetus pregnancies at risk for preterm delivery, i.e., at risk of preterm birth less than 34 weeks of gestation and had no contraindication to antenatal steroids admitted to the labor room of Srirama Chandra Bhanja (SCB) Medical College & Hospital, Cuttack, India. Ethical approval was taken from the Institutional Ethics Committee (ECR/84/Inst/OR/2015/RR-26), and informed consent was taken from the participants. Preterm pain in the abdomen was the main indication for hospital admission and steroid use.

All patients with singleton pregnancy, who are at the gestational age of 28-34 weeks, having risk of preterm delivery, and who have received steroid prophylaxis were included in the study. Meanwhile, patients who are receiving medications, such as magnesium sulfate (MgSO_4_), narcotic analgesics, digoxin, and beta blockers, which might hinder the biophysical profile (BPP) and Doppler studies, have chorioamnionitis, have known major fetal anomaly, are undergoing steroid therapy other than qualifying course active preterm labor at the time of the study, and have psychiatric illness precluding informed consent were excluded.

Methodology

After completion of the baseline examination, the patients were subjected to steroid administration, either with dexamethasone (four doses of 6 mg, 12 hours apart) or betamethasone (two closes of 12 mg, 24 hours apart). An ultrasonogram (USG) study for the BPP score and Doppler assessment of the umbilical artery (UA) and middle cerebral artery (MCA) were performed at 0 and 48 hours after the first dose of steroid administration. All the examinations were carried out in a single machine of the Voluson™ P8 model (GE HealthCare, USA) by one person between 8 AM and 12 noon to control the fetal circadian rhythm. BPP was observed for a period of 30 minutes. Point 0 or 2 was assigned for every component of the BPP, fetal movement, prenatal non-stress test (NST), fetal tone, amniotic fluid volume (AFV), and breathing movements. Fetal heart rate tracing was recorded daily for 30 minutes before the biophysical score was obtained, evaluated, and interpreted as reassuring or non-reassuring, as proposed by the American College of Obstetricians and Gynecologists (ACOG). Doppler studies were performed immediately after obtaining the BPP score. For Doppler, both the UAs and MCA were sampled at the lowest feasible incident angle. The systolic/diastolic (S/D) ratio, pulsatility Index (PI), and resistance index (RI) of both the MCA and UAs were obtained.

Data analysis

Data were collected and entered into Microsoft Office Excel 2007 worksheet (Microsoft Corporation, USA) and analyzed using IBM SPSS Statistics for Windows, Version 20 (Released 2012; IBM Corp., Armonk, New York, United States). Categorical variables were expressed in terms of frequencies and percentages. Continuous variables were expressed in terms of mean and standard deviation (SD).

## Results

Out of 59 eligible cases, 27 (45.8%) were less than 25 years of age, 26 (44.1%) were between 25 and 30 years of age, and six (10.2%) were less than 30 years of age. The range was between 19 and 33 years. The mean age was 25.31 years with an SD of 3.743 (Table 1).

**Table 1:** Age category of the study participants

Age category	Frequency	Percentage (%)
<25 years	27	45.8
25-30 years	26	44.1
>30 years	6	10.2
Total	59	100.0

A total of 96.6% of cases were admitted with a chief complaint of abdominal pain. In the urine culture, only seven (11.9 %) cases came out positive with a urinary tract infection and pathogenic organisms (Table 2).

**Table 2:** Chief complaints at the time of admission

Chief complaints	Frequency	Percentage	Valid percentage (%)
Leaking pervaginum	1	1.7	1.7
Abdominal pain	57	96.6	96.6
Urinary retention	1	1.7	1.7
Total	59	100.0	100.0

The mean of the BPP scores before steroid administration was 9.19 ± 1.23 and decreased to 5.56 ± 1.05 after 48 hours of the first dose of steroid. The p-value was <0.001, which was statistically significant. Based on the comparison of different BPP profiles before and after the administration of steroids, the administration of betamethasone decreased fetal movements by 78.6% and breathing by 71.8%. As a result, the BPP scores decreased significantly (Table 3).

**Table 3:** Comparison of the different parameters of the biophysical profile before and after steroid administration AFI: amniotic fluid index; NST: non-stress test

Components	Before steroid administration		After steroid administration		p-value
	Present n (%)	Absent n (%)	Present n (%)	Absent n (%)	<0.001
Gross body movement	52 (88.1)	7 (11.2)	6 (10.2)	53 (89.8)	
Breathing	53 (89.8)	6 (10.2)	9 (18.0)	50 (82.0)	
Tone	51 (13.5)	8 (86.5)	44 (74.5)	13 (25.5)	
AFI	49 (83.0)	10 (17.0)	47 (79.7)	12 (20.3)	
NST	58 (98.3)	1 (0.7)	43 (72.9)	16 (27.1)	

Based on the comparison of the UA Doppler velocimetry before and after steroid administration, there was a decrease in the mean of all the Doppler indices, but the decrease was not statistically significant (Table 4).

**Table 4:** Comparison of the umbilical artery Doppler velocimetry before and after steroid administration S/D ratio: systolic/diastolic ratio; PI: pulsatility index; RI: resistive Index; SD: standard deviation

Variable	Before steroid (mean+ SD)	After steroid (mean+ SD)	p-value
S/D ratio	2.90 ± 0.30	2.89 ± 0.33	0.657
PI	1.07±0.19	1.00±0.11	0.001
RI	0.66±0.09	0.65±0.06	0.34

Based on the comparison of the MCA Doppler velocimetry before and after steroid administration, there was a decrease in the mean of all the Doppler parameters in the MCA. These decreases in the SD ratio from 3.76± 0.45 to 3.70 ± 0.44 and pulsatility index (PI) from 1.28 ± 0.32 to 1.24 ± 0.28 were statistically significant with p-value < 0.05. However, the decrease in the RI of the MCA from 0.88 ± 0.09 to 0.88 ± 0.07 was not statistically significant (Table 5).

**Table 5:** Comparison of MCA Doppler velocimetry before and after steroid administration MCA: middle cerebral artery; S/D ratio: systolic/diastolic ratio; SD: standard deviation; PI: pulsatility index; RI: resistive index

Variable	Before steroid administration (mean+ SD)	After steroid administration (mean+ SD)	p-value
S/D ratio	3.76 ± 0.45	3.70 ± 0.44	0.018
PI	1.28 ± 0.32	1.24 ± 0.28	0.036
RI	0.88 ± 0.09	0.88 ± 0.07	0.505

The cerebroplacental ratio (CPR) value in our study was 1.24, more than 1.08, suggesting that the decrease in Doppler indices in UA and MCA after 48 hours of steroid administration is not clinically important.

## Discussion

This study was performed on 59 singleton pregnant women experiencing preterm labor pain with a mean age of 25.31 + 3.7 (19-33 years). The mean gestational age of admission was 31.37 + 1.87 (28-34 weeks), and most (98.3 %) were admitted with preterm labor pain. These patients were administered one course of corticosteroids, either with dexamethasone or betamethasone, to facilitate pulmonary maturation. Doppler assessment and fetal BPP were performed twice first on hospital admission and 48 hours after administration of the first dose. The outcome of the BPP and Doppler indices of the UA and MCA before and after corticosteroid administration were compared. The BPP before and after corticosteroid administration was assessed and compared, and the results showed that the mean BPP score decreased significantly within 48 hours of corticosteroid administration. Rotmensc et al. studied two groups of women in preterm delivery exposed to dexamethasone and betamethasone, and the results were observed 48 hours after injection [10]. According to the study, gross fetal and thoracic movements decreased in 50.9% and 86.1% of the participants, respectively [10]. The studies done by Tehrani et al. also reported the same results [11]. Jackson et al. also observed the same decrease in fetal movements and breathing motion after corticosteroid administration [12].

The result obtained in our study shows that gross fetal movements and fetal breathing movements were decreased in 78.6% and 71.8% of the participants, respectively. Some studies have related the amniotic fluid index (AFI) changes to corticosteroid injection. In Jackson et al.'s study, the AFI decreased exactly after corticosteroid injection in about 72% of their sample size [12]. According to the study done by Kazardoost et al., betamethasone injection was considered a reason for the AFI decrease [13]. However, contrary to our study results, although AFI was decreased (3.3 %) in some cases, no significant influence was observed owning to corticosteroid injection. According to Mulder et al.'s study, only short and long beat-to-beat indices were decreased due to corticosteroid injection, with no effect on acceleration [14]. Our study only observed non reassuring NST in 26.4% of the samples.

The mechanism that suppresses the biophysical activities of steroids is not clearly defined. The ability of synthetic glucocorticosteroids to suppress neural activities has been researched [15]. The glucocorticoid receptors are present in the cerebral cortical tissues, midbrain, and subcortical nuclei, which may partly explain the suppression. Some dexamethasone preparations contain a sulphite preservative (Neonatal Formulary 5 (NNF5) 2006), which are linked to neurotoxicity in newborns, especially in combination with peroxynitrite [16]. Another method for the evaluation of the fetal status is Doppler velocimetry of the umbilical and cerebral circulation. The studies done by Dawes et al. showed that the antenatal corticosteroids did not affect Doppler indices obtained from the fetal, placental, or uterine arteries [17]. Kazardoost et al. concluded that the MCA and UA Doppler indices decreased after antenatal steroid administration [13]. Pooransari et al. published the results of the effect of betamethasone on fetal circulation in preterm fetuses, wherein the reduction of the UA PI (p = 0.025) and RI (p = 0.019) were statistically significant [18]. The results obtained in our study show that after antenatal corticosteroid administration, there is a decrease in the Doppler indices of the UA and MCA after 48 hours. The UA PI before the steroid administration was 1.07 + 0.19 and after the steroid administration was 1.00 + 0.11 with a p-value of 0.001. The MCA PI before the steroid administration was 1.28 + 0.32 and after the steroid administration was 1.24 + 0.28 with a p-value of 0.036. Although the values are statistically significant, these changes are not important clinically, as the CPR, i.e., PI of the MCA to PI of the UA, is 1.24 (>1.08).

The outcome of this research depicts this modality's reliability for assessing fetuses previously exposed to antenatal steroids. Doppler studies can differentiate steroid-induced changes in the fetal BPP from those due to fetal compromise. The rise in antenatal steroid usage increases the rate of abnormal BPPs. This is an evident matter for notable clinical consideration. These transient changes often result in unwarranted iatrogenic preterm delivery. The limitation of this study was the small sample size. Hence, further research is required, which involves a large sample size that would provide a better concept in this specified area of research.

## Conclusions

Preterm birth (< 34 weeks) is the major cause of perinatal morbidity and mortality globally, and its prevention is an essential healthcare priority. Preterm parturition is one of the "great obstetrical syndromes." One of its major complications is respiratory distress syndrome, which can be prevented by using prophylactic maternal corticosteroids in women at risk of preterm delivery. The rise in antenatal steroid usage leads to an increase in the rate of abnormal biophysical profiles. Doppler indices are essential in differentiating between steroid-induced compromise in the fetus and real fetal distress.
